# In Vivo Magnetic Resonance Spectroscopy Methods for Investigating Cardiac Metabolism

**DOI:** 10.3390/metabo12020189

**Published:** 2022-02-18

**Authors:** Morteza Esmaeili, Riyas Vettukattil

**Affiliations:** 1 Department of Diagnostic Imaging, Akershus University Hospital, 1478 Lørenskog, Norway; 2 Department of Electrical Engineering and Computer Science, Faculty of Science and Technology, University of Stavanger, 4021 Stavanger, Norway; 3 Faculty of Medicine, Institute of Clinical Medicine, University of Oslo, 0315 Oslo, Norway; m.r.vettukattil@medisin.uio.no; 4 Division of Paediatric and Adolescent Medicine, Oslo University Hospital, 0372 Oslo, Norway

**Keywords:** heart failure, bioenergetics, creatine kinase, metabolic disorders, oxidative metabolism

## Abstract

Magnetic resonance spectroscopy (MRS) is a non-invasive and non-ionizing technique, enabling in vivo investigation of cardiac metabolism in normal and diseased hearts. In vivo measurement tools are critical for studying mechanisms that regulate cardiac energy metabolism in disease developments and to assist in early response assessments to novel therapies. For cardiac MRS, proton (^1^H), phosphorus (^31^P), and hyperpolarized 13-carbon (^13^C) provide valuable metabolic information for diagnosis and treatment assessment purposes. Currently, low sensitivity and some technical limitations limit the utility of MRS. An essential step in translating MRS for clinical use involves further technological improvements, particularly in coil design, improving the signal-to-noise ratios, field homogeneity, and optimizing radiofrequency sequences. This review addresses the recent advances in metabolic imaging by MRS from primarily the literature published since 2015.

## 1. Myocardial Metabolism in Failing Heart

The heart is one of the most energy-consuming organs in our body. To tackle its high energy demand, heart muscles (myocardium) have a complex and efficient metabolic machinery for high energy phosphate and adenosine triphosphate (ATP) production from various energy substrates, such as fatty acids, carbohydrates, and ketones. The heart muscles are highly flexible in their energy metabolism and quickly adapt to the growing energy demand. In heart failure (HF) disease, the heart muscles cannot adequately pump and circulate blood through the body. Moreover, there is a loss of flexibility in the myocardial energy metabolism, which results in impaired ATP production. HF is continuously evolving, with an estimation of over 25 million diagnosed patients globally [1]. This complex disease is fatal and poses a significant socio-economic burden to the healthcare system [2,3]. Heart failure is diagnosed by a spectrum of symptoms. The heart cannot maintain regular contractions, harmonic systolic and diastolic movements, and overall circulation functionality. Metabolic remodeling may serve as a signature of a failing heart.

The term “engine out of fuel” is often used in the literature to precisely describe the interplay between failing heart and bioenergetics dynamics [4]. The failing heart cannot compensate for the increased energy demands due to less efficient energy metabolism [5]. Dysregulated metabolism may also be extended to skeletal muscles with similar bioenergetics starvation symptoms. Together, these shortages in biosynthetic metabolism contribute to the deterioration of patients’ general health and mobility, experiencing muscle weakness, fatigue, exercise limitation, and dyspnea to disease progression. Magnetic resonance spectroscopy (MRS) is the only in vivo method that allows cardiac metabolism investigations non-invasively. Despite the complications of performing clinical MRS, translating the technique into clinical cardiac examination is not impossible, as described in this paper.

## 2. ATP and Phosphocreatine

**Figure 1:**
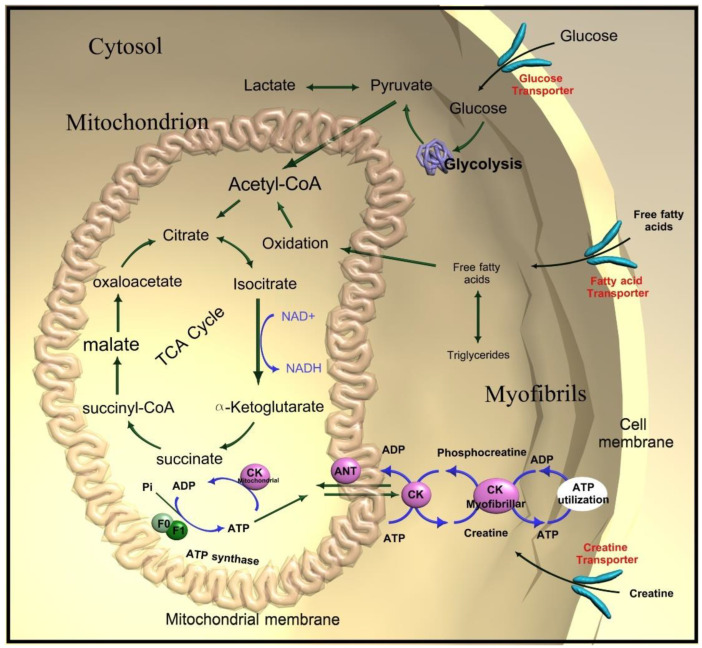
Creatine kinase (CK) energy shuttle. ADP—Adenosine diphosphate; ATP—adenosine triphosphate.

The most energy-consuming reactions in the heart, such as the contraction of the myofilaments of the active pump function, are fueled by ATP utilization. ATP is partly obtained from phosphocreatine (PCr) via the creatine-kinase (CK) process occurring in the mitochondria (CK_mitochondrial_) (Figure 1). The heart uses PCr as the prime temporal and spatial energy reserve [6]. PCr transports energy within the cell and acts as a short-term energy buffer, which helps in the rapid regeneration of ATP at increased demand. Mitochondrial dysfunction leads to myocardial energy depletion by poor energy transfer to the myofibrils and impaired uptake and use of ATP and PCr [7]. The enzyme CK_mitochondrial_ helps transfer ATP’s gamma phosphoryl group to creatine, resulting in the formation of PCr. A reverse reaction follows with a transfer of the phosphoryl group to adenosine diphosphate (ADP) to generate ATP. A reduction in the activity of the CK system is a hallmark of heart failure regardless of etiology [8]. New approaches in measuring altered energetics may be useful in timely detection and monitoring HF.

At rest, free fatty acid oxidation provides the primary energy source for the myocardium [9]. The oxidation of free fatty acids produces up to 60 to 80% of high-energy phosphates at rest [9]. Studies of cardiac metabolism show that in HF, there is a change in the proportion of fatty acid and glucose uptakes and utilization [7]. Unhealthy heart muscles tend to increase the glucose substrate, switching from fatty acids metabolism towards glucose [10,11]. Furthermore, the failing heart increasingly relies on cytosolic glycolysis, reducing mitochondrial fatty acid and glucose oxidation [10,11]. This reprogramming may be interpreted as the heart’s reduced metabolic flexibility and dynamics and a shift towards a single energy source.

## 3. MR Spectroscopy

Magnetic resonance (MR) signals arise from the intrinsic magnetic moments of specific atomic nuclei. MR imaging (MRI) and MR spectroscopy detect nuclides with an odd number of protons and neutrons. Protons (^1^H) are the most commonly utilized nuclide due to their high intrinsic sensitivity and ~100% natural abundance in the human body. Others include ^31^P phosphorus, ^13^C carbon, and ^19^F fluorine, which have been well-established and examined.

The nuclear magnetic moments (spins) align in an MRI scanner, precessing at a particular resonance frequency, called the Larmor frequency (in MHz). This frequency is directly proportional to the strength of the externally applied magnetic field (in Tesla) and a constant gyromagnetic ratio for a specific nucleus. The Larmor frequency varies for every nuclide (e.g., the gyromagnetic ratio of ^1^H is 42.58 MHz/Tesla, and for ^31^P is 17.24 MHz/Tesla). The aligned spins produce a net magnetization pointing toward the scanner’s main magnetic field direction, referred to as B_0_. This equilibrium magnetization can be perturbed by applying a radiofrequency (RF) “excitation pulse” at the Larmor frequency—the nuclei absorb this RF energy, causing the magnetization to tilt away from the B_0_ (B_0_ direction is commonly designated along the Z-axis). The magnetic field component of the electromagnetic RF pulse is referred to as B_1_. Conceptualizing the net magnetization as a rotating vector, and at the equilibrium state, this vector is aligned to B_0_ (longitudinal magnetization, along Z direction). Applied 90-degree RF pulse tilts the energized vector to the XY plane, producing a component perpendicular to the B_0_ (transverse magnetization). The “reception pipeline” could be a separate surface coil or integrated with the “transmission pipeline” in a volume coil. The hardware accomplishes the switching between transmission to receiving. Tuning the RF mode from a transmitter to a receiver detects the RF energy by the RF receiver coil elements tuned to the Larmor frequency of the excited nuclei.

Nuclei within a molecule experience slightly different magnetic fields based on molecular environments, leading to chemical shielding and chemical shift alterations for the otherwise identical nuclei in the molecule. Therefore, nuclei within the molecule precess at slightly different frequencies. The resonance offsets can be described on a field-independent dimensionless scale called chemical shift (δ), expressed in dimensionless metric parts per million (ppm)—which is normalized to the main B_0_ field (see reference [12]). Using ppm scale, the chemical shift of a specific metabolite (molecule) will be expressed at the same position in the MR spectrum regardless of the acquisition with different MR systems and field strength. For example, in a proton spectrum, methyl groups resonate around 1.3 ppm, and water resonates at 4.7 ppm.

Cardiac MRS enables an in vivo detection and quantification of myocardial metabolism, examining healthy and pathological conditions. It is possible to acquire MRS data derived from different endogenous nuclei (Table 1). Phosphorus is the most widely investigated for myocardial bioenergetics studies. Additionally, proton MRS can visualize crucial molecular triglycerides and creatine (Cr). MR spectra can be acquired from single volume elements (single-voxel spectroscopy (SVS)) with localization determined from MR images or from multiple volume elements (multi-voxel), which is referred to as multi-voxel magnetic resonance spectroscopic imaging (MRSI) or chemical shift imaging (CSI) [13,14]. In MRS, individual metabolites are detected based on their resonant frequencies that can be encoded and identified. This technique allows the acquisition of MR spectra simultaneously from a two-dimensional (2D) or three-dimensional (3D) array of voxels. 2D MRSI extends the SVS to a selective slice, in which the in-plane voxels are phase-encoded. 3D MRSI is further extended to a volume to increase the metabolic mapping coverage. Anatomical MR images, usually navigator-gated balanced steady-state free precession cine images, are used as a guide to planning the MRSI grid (voxels) (Figures 3 and 4). This section provides examples from recently published articles and a brief overview of the possible interaction between MR Spectroscopy and clinical practice.

## 4. Proton Spectroscopy

**Figure 2:**
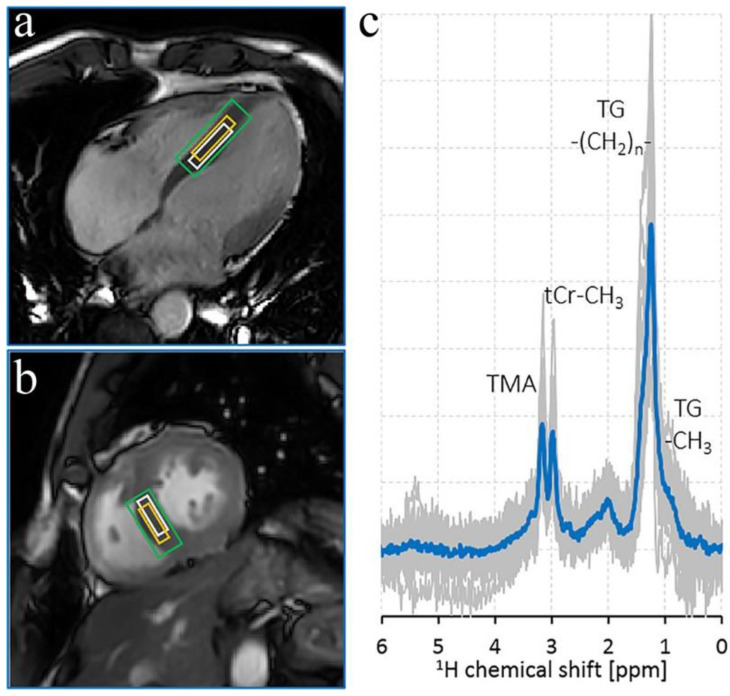
^1^H MR spectrum from the myocardium of a volunteer. The left panel displays the voxel positioning. (**a**) A quasi-4-chamber view (**b**) and an end-systolic short-axis view. (**c**) The right panel depicts the MR spectra (mean spectrum in blue). TMA, trimethylamine-containing compounds; tCr-CH_3_: total creatine-methyl; TG–CH_2_–)_n_ triglyceride-methylene. Adapted from ref. [15] an open-access article under the terms of the Creative Commons CC BY license.

A dominant peak in ^1^H MR spectra is total creatine (tCr: creatine + phosphocreatine) resonance at 3.0 ppm (Figure 2) [15]. ^1^H MRS can detect lipids and macromolecules around 0.9–2.5 ppm (Figure 2). Myocardial triglycerides and trimethylamine-containing compounds (TMA) are also detected by this technique [15,16]. TMA is oxidized by hepatic flavin monooxygenase 3 in the liver and forms TMAO, generally identified as a pathogenic metabolite leading to hypertension [17]. Several studies have investigated the link between increased plasma TMAO and heart failure, revealing that TMAO concentration is associated with advanced left ventricular diastolic dysfunction and poorer clinical outcomes in heart failure patients [17]. The relatively increased concentration of lipids is usually associated with, among several malfunctions, type 2 diabetes mellitus and impaired diastolic function [18]. However, several studies have addressed the complexity of distinct evaluation of intra-myocellular lipids (IMCL) from extramyocellular lipids (EMCL) [16,19]. Precise detection of IMCL and EMCL compartments is essential to investigate lipid metabolism dynamics in pathological development [16].

Several acquisition developments have been introduced to enable cardiac ^1^H MRS [20,21]. These techniques include rapid single-breath-hold acquisitions, prospective volume-tracking respiratory gating with pressure transducers, navigator techniques, and improved B_0_ field homogeneity by advanced high-order shimming. Cardiac MR and MRS are usually obtained with routine electrocardiogram (ECG) triggering [22], alternative triggering methods [23], or navigator-free approaches [24]. Post-processing developments have also contributed to the quality of ^1^H MR spectra, including frequency alignment, phase correction, phase-cancellation derived from cardiac motion, B_0_ and B_1_ mappings to compensate for inherent inhomogeneities [25]. In ^1^H cardiac MRS, the remaining un-suppressed signal from water contributes to frequency alignment and phase correction [19]. Furthermore, the water signal guides the smooth use of ECG navigations and navigator echoes to maintain respiratory gating and reduce respiratory motion artifacts. Unsuppressed or well-suppressed water approaches include a two-shot subtraction technique where the water peak has retained a reference for spectral correction [16].

## 5. Phosphorus Spectroscopy

**Figure 3:**
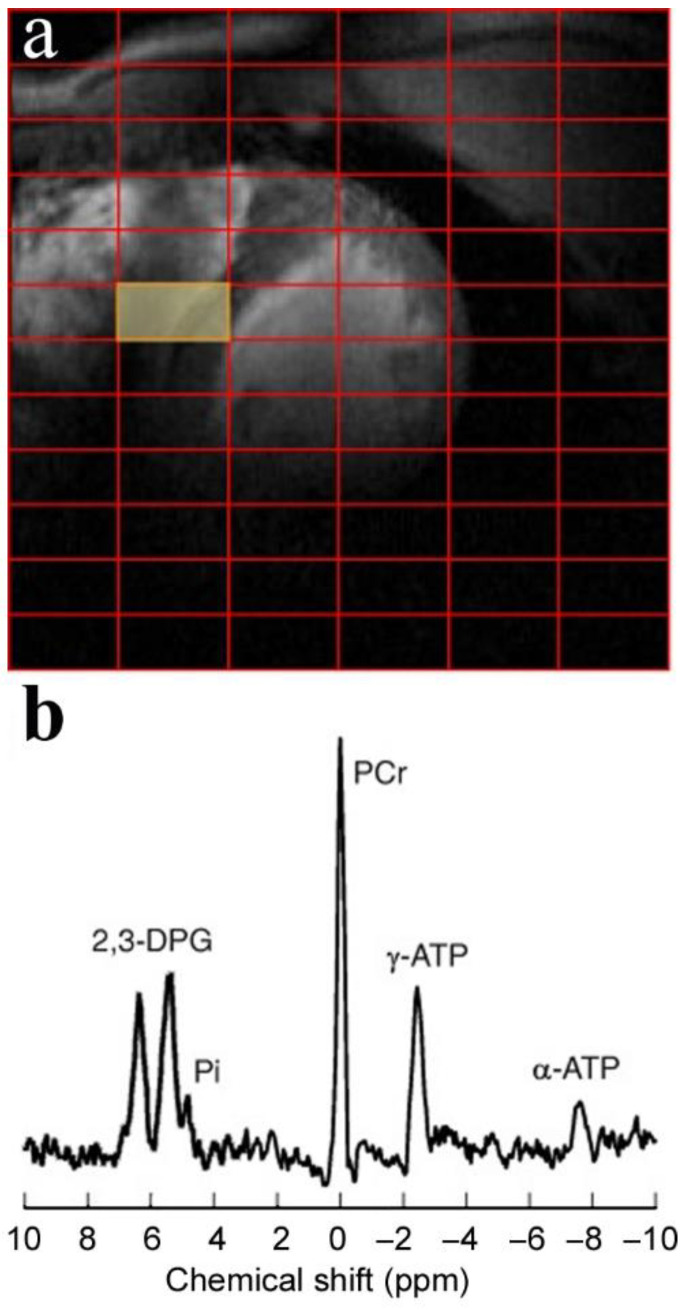
Cardiac ^31^P MRSI at 7 Tesla. (**a**) Localizer images: positions of the MRSI grid on the short-axis view of the heart. (**b**) MR spectra from the corresponding colored voxels marked on the localizer images. The spectra were acquired in a healthy subject using a 3-dimensional ultra-short-echo CSI sequence, matrix size of 8 × 16 × 8, nominal voxel size of 30 × 15 × 25 mm^3^, and five averages (for more details on the CSI protocol, see [26]). The illustration is adapted from [26], an open-access article under the terms of the Creative Commons CC BY license.

Despite a ~100% natural abundance in the human body, ^31^P MRS has a lower intrinsic sensitivity than proton spectroscopy. The gyromagnetic ratio of ^31^P is 17.24 MHz/Tesla—one/third that of the ^1^H. However, the number of phosphorus-containing MR detectable metabolites in the body using phosphorus spectroscopy is more than the proton. ^31^P MRS directly quantifies biochemical of cardiac high-energy phosphate metabolites, such as PCr and ATP, inorganic phosphate (Pi), and other phosphorus-containing metabolites, such as phosphomonoesters (PME) and phosphodiesters (PDE). The PME consists of phosphocholine (PCho) and phosphoethanolamine (Petn). The PDE includes glycerophosphocholine (GPC) and glycerophosphoethanolamine (GPE). The major peaks detected by ^31^P cardiac MRS are ATP, PCr, Pi, and 2,3-diphosphoglycerate (2,3-DPG) (Figure 3). Furthermore, the technique can non-invasively measure intracellular pH (pHi) [26]. The pHi measurements rely on the chemical shift difference between pH-dependent Pi resonance and pH-independent, in the physiological range, PCr or α resonance of ATP (ATP_α_) resonances [27].

When ATP demand rises, a healthy body will trigger diversely interrelated and coupled physiological processes to stimulate more ATP productions. However, unhealthy bodies, such as ischemic myocardium, HF, diabetes, and obesity lead to impaired energy production capacity; thus, the PCr level decreases remarkably to maintain ATP levels. The ATP signal only starts to fall by a significantly reduced PCr level [4]. As the ATP concentrations remain steady, the alteration in PCr/ATP ratio may primarily reflect the decline in PCr. This assumption has been demonstrated in several ^31^P cardiac studies [28,29,30,31], as the PCr/ATP ratio changes may dominantly imply a fall in PCr concentration and an increase in ADP to maintain ATP/ADP and PCr/Cr equilibrium. Some technical solutions, such as using a known concentration of phosphorus metabolite as an external reference, may enable the absolute quantification of PCr and ATP [32,33].

**Figure 4:**
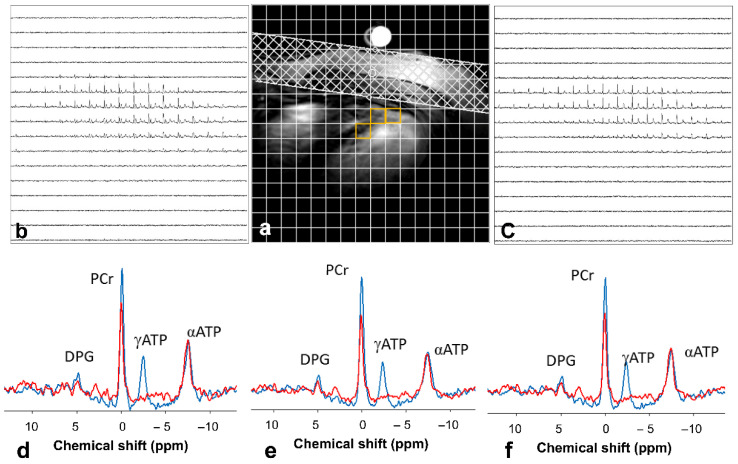
Saturation transfer techniques allow CK examinations in vivo. (**a**) An anatomical image highlights the typical voxel (orange) location chosen for analysis. ^31^P CSI from the slice (**b**) control and (**c**) saturation conditions. (**d**–**f**) Typical ^31^P magnetization transfer spectra from selected voxels. Control spectra are shown in blue and saturated spectra (γATP saturation) are in red. The PCr signal is reduced when γATP is saturated. Illustration from [35] an open-access article under the terms of the Creative Commons CC BY license.

Furthermore, ^31^P MRS provides critical insight into energy turnover by an indirect unidirectional CK flux and ATP synthesis measure. The conventional ^31^P saturation transfer techniques [34] allow quantification of CK reaction rate to study ATP transition from mitochondria for cellular consumptions. Using frequency-selective saturation pulses [35,36], saturation transfer methods suppress the ATP’s gamma phosphoryl group (ATP_γ_), resulting in a decreased signal PCr [35,36] (Figure 4). The method enables an estimate of the kinetic rate for the exchange of ATP to PCr [35,36].

By allowing non-invasive molecular investigations, MR spectroscopy has contributed significantly to understanding cardiac metabolism in HF under physiologic conditions [6,37,38]. The cardiac PCr/ATP ratio level provided a sensitive biomarker in HF patients [6,38]. Some studies have demonstrated a significant correlation between decreased PCr/ATP and systolic HF and dilated cardiomyopathy (DCM) [39,40] and impaired peak systolic circumferential strain [41] at rest. PCr/ATP has been used as a sensitive metric to assess and evaluate exercise training and novel treatment on HF or coronary artery disease patients [41,42]. ATP flux and CK reaction investigations have demonstrated a significant reduction in HF patients and other cardiac diseases, such as non-ischemic DCM and left ventricular hypertrophy [34]. Also, HF diseases induce extensive energetic abnormalities, derangements in high energy phosphate metabolism, reduced CK flux, and lipid accumulation. Cardiac PCr/ATP was shown to correlate with the New York Heart Association functional class of symptom severity in DCM and HF patients [4]. Conventional medical treatments, such as b-blockers—angiotensin-converting enzyme inhibitors—have increased PCr/ATP levels [43,44]. Thus, bioenergetic abnormalities are not specific markers to HF and may indicate other myocardium pathologies and subsequent adverse cardiovascular outcomes.

The most critical elements impeding the clinical application of the ^31^P MRS technique are low sensitivity and lack of specificity. Additionally, the spatial resolution limits the application of ^31^P MRS for heterogeneous diseases, such as myocardial infarction. The sensitivity of spectroscopic acquisitions has improved in the past years due to the better performance of modern digital receivers. Cardiac ^31^P MR spectroscopy may significantly benefit from advanced coil designs, such as the inclusion of phased arrays and dynamic shimming capability [45,46]. Additionally, acquiring at ultra-high-field strength (≥7 Tesla) increases the MRS sensitivity, accelerates data acquisition, and allows higher spatial resolution.

However, overcoming the technical limitations of ^31^P MRS is not the only driver of clinical usage. ^31^P MR spectroscopy has not yet provided a practical clinical application to justify multinuclear capability on the clinical systems. Without the necessity to implement such diagnostic imaging examinations, insurance companies will not cover the related expenses. Thus, on the institutional level, the financial investments on multi-nuclei coils and examinations are relatively less prioritized for research if they are purely clinical sites. Consequently, clinical MR vendors have demonstrated a little momentum to develop ^31^P coils and examinations in general as the market is limited.

## 6. Carbon Spectroscopy

^13^C MRS can be used to non-invasively investigate metabolic processes, such as the TCA cycle, glycolysis, gluconeogenesis, ketogenesis, and ethanol metabolism [47]. The application of ^13^C in the clinical setting has been limited by intrinsically low sensitivity (Magnetically active carbon, ^13^C, only has a 1.109% natural abundance). Additionally, the ^13^C gyromagnetic ratio is 10.705 MHz/Tesla, four times lower than the proton. However, development in the use of hyperpolarization using the dynamic nuclear polarization (DNP) technique, which is a process that can enhance >10,000-fold signal increases in MR-active nuclei, has improved the prospects [48]. Hyperpolarized ^13^C MRS enables a quantitative assessment of the pyruvate dehydrogenase complex flux, which converts pyruvate to acetyl-coenzyme A (acetyl-CoA), one of the vital metabolic processes substrates altered in a failing heart [49,50]. The rapid metabolism and relatively short T1 relaxation times of most biologically relevant hyperpolarized ^13^C probes are limitations while studying a slower biological process.

It is technically possible to assess the changes in cardiac mitochondrial function, especially a reduction in TCA cycle (Krebs cycle) activity in a post-myocardial infarcted heart using hyperpolarized ^13^C MRS using labeled pyruvate. The carbon-13 from pyruvate is incorporated into downstream metabolites, which hyperpolarized ^13^C MRS can detect these metabolites. The changes in signal intensity of pyruvate compared to that of the downstream metabolites in a certain time window can be used to determine kinetic information about the TCA cycle (for a graphical overview, see ref. [51]) [52]. For example, the pyruvate oxidation pathway can be explored by tracing the ^13^C labeled throughout the involved pathways’ enzymes: lactate dehydrogenase, pyruvate dehydrogenase complex, and alanine aminotransferase. Some studies have proposed kinetic models to quantify the substrate conversion rate constants [53,54]. The model provides the exchange rate between two substrates detectable by ^13^C MRS; for example, the exchange rate of pyruvate to lactate identifies the k_PL_ constant [53,54]. Other potential valuable molecules are short-chain fatty acids ([1-^13^C] acetate and [1-^13^C] butyrate), which can be used to trace the fatty acid metabolism in the heart [55]. Also, hyperpolarized [1-^13^C]pyruvate has been employed to generate ^13^CO(2) and H^13^CO(3)(-) measuring cardiac pHi in vivo [56]. Recent studies have also demonstrated that absolute myocardial blood flow quantification using hyperpolarized [1-^13^C]pyruvate is feasible in an in vivo setting [57,58].

## 7. Coil and Dynamic Shimming Approaches

MR imaging and spectroscopy acquisitions will benefit from ultra-high-field examinations [59]. However, the static magnetic field (B_0_) inhomogeneities almost increase linearly with the field strength, leading to new challenges in cardiac magnetic resonance studies at 7 Tesla [59]. Some intrinsic physiological sources that perturb the B_0_ field include (i) distinct MR susceptibility properties arising from the myocardium, blood, and surrounding tissues, (ii) geometrical shape of the heart, and (iii) beating heart and circulation-related motions [45,59]. Furthermore, fast data sampling techniques like spiral readout trajectories and echo-planar imaging are adversely affected by field inhomogeneity.

The amount of power deposited by a radiofrequency field in a certain mass of tissue must be limited to minimize patient heating, which puts additional constraints on the imaging sequences. Several recent studies have proposed novel solutions to effectively homogenize the main B_0_ magnetic field (referred to as shimming) locally in the heart [45,46]. Several methods have also been developed to optimize the B_1_ field and RF transmission. Some studies have demonstrated the impact of higher-order shimming protocols and parallel transmit and receive RF coils, where instead of a single-coil, separate independently powered and controlled coil elements are used to improve the RF excitation and reception on the human brain at 3 Tesla and 7 Tesla [45,46,59,60,61].

Shim coils integrated inside the MR systems and around the scanner’s bore correct the distribution of the static field. Defining a shim box on a volume of interest can also achieve more refined shim corrections. Clinical MR systems shim coils generate magnetic fields up to what so-called first and second-order shims can be used to improve the B_0_ field. The acquired B_0_ field map is used to adjust the shim prior to running the scans. Usually, the 1st–2nd-order shimming is used in correcting B_0_-field inhomogeneity over the entire region of interest. The higher-order shimming (>2nd order) may significantly benefit the shimming outcomes [62].

**Figure 5:**
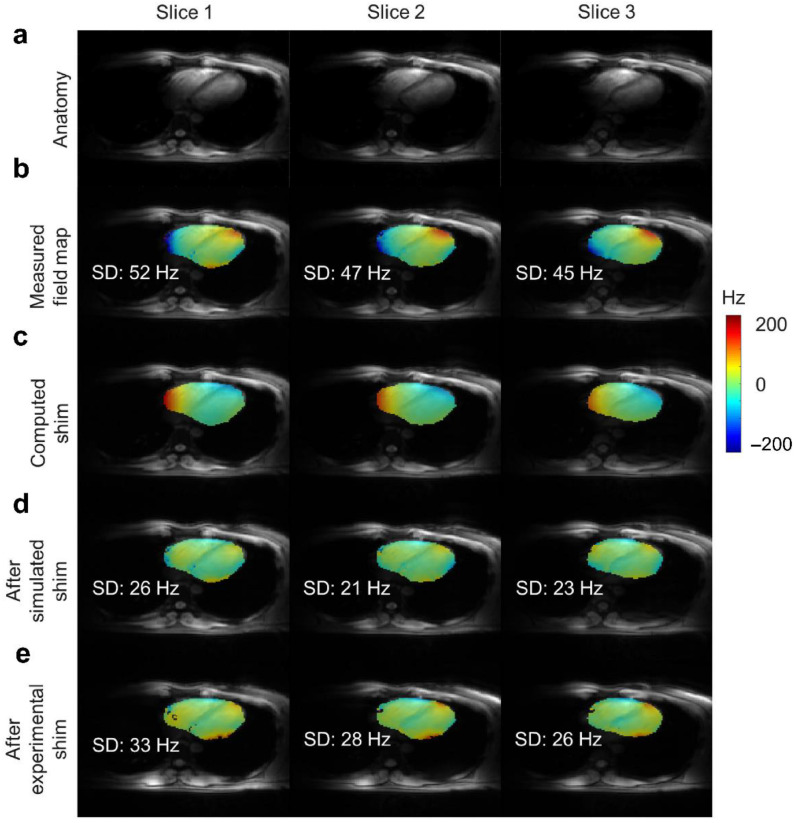
In vivo B_0_ shimming of the heart using 3rd-order sets at 7 Tesla. (**a**) Region of interest on the magnitude images, (**b**) Initial measured B_0_-field distribution (SD, standard deviation, across the sliced region of the heart); (**c**) Computed magnetic shim field; (**d**) Expected B_0_-field distribution after the simulated shim; (**e**) B_0_-field distribution measured after the experimental shim. Illustration is from ref. [45] an open-access article under the terms of the Creative Commons CC BY license.

MR engineers seek new hardware developments to combine a dynamic shimming system with traditional static shimming [63]. New solutions for more advanced shimming have resulted in engineering dynamic and real-time shimming approaches. These techniques include high-order shimming and multi-shim-coil architecture to leverage spatial field homogeneity. Some experimental studies demonstrated significant B_0_ field homogeneity improvements in the heart using anatomy-driven shim region of interest and the vendor-supplied higher-order shimming [45,64] (Figure 5). Also, slice-by-slice (slab-selective compared to the volume shim) shimming approaches have greatly improved the utility of parallel transmission with improved homogenous B_1_ field across the slabs in the heart while decreasing the SAR level [65,66]. These studies aim to eventually include dynamic shim updating on the selected volume in the heart, as has been experimented with within the brain studies at ultra-high-field ≥7 Tesla [67,68]. Overall considerations on the heart shimming include: (i) using slab-selective approaches shimming to minimize the field distribution variations (shimming standard deviation (SD)), (ii) optimizing high-order shims, (iii) temporal B_0_-field variations with regards to cardiac cycle and respiration-induced, (iv) shimming of oblique orientations, (v) develop dynamic shimming interface by rapid field mapping using slab-specific or cardiac phase-specific mapping sets, and (vi) the emerging use of artificial intelligence in compensating the field variations [45,63,66,69,70,71].

## 8. Summary

MR spectroscopy is one of the few currently available techniques for in vivo metabolic imaging, enabling in vivo assessments of cardiac energy metabolism and mitochondrial functions. Although MRS has a promising potential for identifying biomarkers for disease and evaluating treatment response, the spectroscopic appearance of many pathologies can overlap. While ^31^P MRS has been primarily used in measuring cardiac high-energy phosphates and turnover, the emerging use of other MRS techniques provides significant progress. Studies on both HF animal models and clinical examinations have led to several findings in the field. The utility of MRS has been limited to research institutions because of several technological demands and requirements. In particular, ^31^P and hyperpolarized ^13^C MRS studies are investigated in very few research centers. To probe and understand the malfunction of cardiac metabolism and metabolic reprogramming towards disease developments, in vivo measures are critical tools. A robust MRS may critically explore the metabolic impairment and bioenergetics instability linked to heart failure developments. Also, an in vivo technique may assist early response to novel therapies. An essential step in translating MRS to clinical use involves further technological improvements, particularly in coil design, improving the signal-to-noise ratios, field homogeneity, optimizing radio-frequency sequences, and kinetic modeling of ^13^C. Other challenges include artifacts from cardiac movements, blood flow, and the effect of the nearby lung. Another crucial element is the improvement in MR acquisition and reconstruction methods. Much work remains to be carried out for these methods to realize their full potential in elucidating cardiac metabolism.

## Figures and Tables

**Table 1:** Detectable nuclei with magnetic resonance spectroscopy techniques and their use in cardiac MR spectroscopy.

Nuclei	Metabolites	Significance
Proton (^1^H)	Creatine, triglyceride, lipid	Lipid and creatine pools within the myocytes
Phosphorus (^31^P)	Adenosine tri phosphate (ATP); phosphocreatine (PCr); PCr/ATP ratio, pHi	Bioenergetics metabolites, intracellular pH measurements
Carbon (^13^C)	Glucose, lactate, pyruvate, fatty acids, lipid	Probe TCA cycle metabolism, pyruvate dehydrogenase, glycolysis, fatty acid metabolism
